# Experimental Investigation of the Influence of the Laser Beam Waist on Cold Atom Guiding Efficiency

**DOI:** 10.3390/s18030717

**Published:** 2018-02-28

**Authors:** Ningfang Song, Di Hu, Xiaobin Xu, Wei Li, Xiangxiang Lu, Yitong Song

**Affiliations:** Department of Instrumentation Science and Opto-electronics Engineering, Beihang University, Beijing 100191, China; Songnf@buaa.edu.cn (N.S.); hudi17@buaa.edu.cn (D.H.); li.wei@buaa.edu.cn (W.L.); luxiangxiang@buaa.edu.cn (X.L.); songyitong@buaa.edu.cn (Y.S.)

**Keywords:** cold atom guiding, beam waist, guiding efficiency

## Abstract

The primary purpose of this study is to investigate the influence of the vertical guiding laser beam waist on cold atom guiding efficiency. In this study, a double magneto-optical trap (MOT) apparatus is used. With an unbalanced force in the horizontal direction, a cold atomic beam is generated by the first MOT. The cold atoms enter the second chamber and are then re-trapped and cooled by the second MOT. By releasing a second atom cloud, the process of transferring the cold atoms from MOT to the dipole trap, which is formed by a red-detuned converged 1064-nm laser, is experimentally demonstrated. And after releasing for 20 ms, the atom cloud is guided to a distance of approximately 3 mm. As indicated by the results, the guiding efficiency depends strongly on the laser beam waist; the efficiency reaches a maximum when the waist radius (*w*_0_) of the laser is in the range of 15 to 25 μm, while the initial atom cloud has a radius of 133 μm. Additionally, the properties of the atoms inside the dipole potential trap, such as the distribution profile and lifetime, are deduced from the fluorescence images.

## 1. Introduction

Over the past several years, there has been a growing interest in the transferring and guiding of atoms by the dipole potential trap. The development of efficient atom guiding and manipulating systems is of great importance for applications related to the transportation of cold atoms from the atom source to experimental regions, such as atomic interferometers [1,2], atom optics [3], and atom guiding in hollow-core fibers [4,5].

The concept of guiding cooled atoms by a red-detuned laser beam was theoretically proposed in 1978 [6]. The first experimental demonstration of the trapping and manipulating of atoms by the dipole force was implemented in 1986 [7]. By combining the magneto-optical trap (MOT) with a far-off resonance potential trap, an atomic fountain was guided by a far-off resonance laser in 1999 [8]. In general, the process of atom guiding actually includes two steps; the first step involves transferring cold atoms from an MOT to a dipole trap, and the second consists of guiding the transferred atoms for certain distances [9]. The following studies were conducted to investigate the relative parameters to improve the efficiency of initially transferred atoms in the optical dipole potential, such as the intensity and detuning of the laser beam [10,11]. However, the influence of the guiding laser beam waist on the guiding efficiency of cold atoms was not investigated thoroughly through experiments but rather through theoretical calculations. Using a focused red-detuned Gaussian laser beam, Pruvost et al. [12] made a comparison when the guiding laser was on and off after the atom cloud freely expanded, and found that about 40% of the atoms were transferred to the dipole trap when the laser was on. According to their preliminary calculations, the maximum guiding efficiency would be obtained when the dimensions of the laser and atom molasses were similar. Then, Wolschrijn et al. [13] carried out a follow-up analysis and reported that, based on the fixed atom cloud size and temperature, the depth of the guiding potential was a significant factor influencing the guiding efficiency in addition to the laser beam waist, and the optimal value of the laser beam waist would increase with the depth of the dipole trap.

The two studies mentioned above mainly concentrated on the theoretical analysis and calculation of the influence of the guiding laser beam waist. However, an experimental demonstration, to the best of our knowledge, has not been performed since then. To experimentally verify the influence of the laser beam waist on the guiding efficiency, a cold atom cloud of ^87^Rb was captured in a red-detuned Gaussian beam in this study. By varying the magnification of the beam expander, the effects of the laser beam waist on the atomic guiding efficiency were investigated. Additionally, the analysis of some other characteristics, such as atom cloud temperature, guiding trap lifetime, and effect of laser power, are presented in this report.

## 2. Experimental Principle and Setup

**Figure 1:**
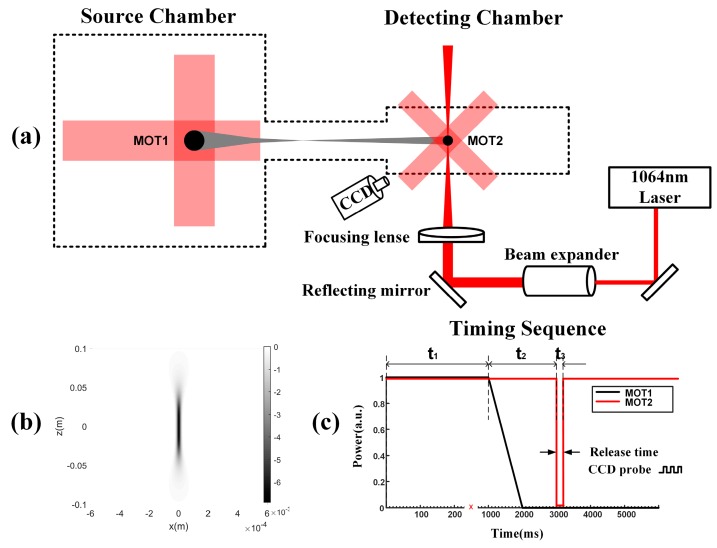
(**a**) Schematic of the experimental setup. (**b**) Simulation diagram of the dipole potential of the guiding laser. (**c**) Timing sequence of experimental procedures in two chambers.

An experimental setup was established to guide the released cold atom cloud of ^87^Rb using a red-detuned Gaussian beam, as illustrated in Figure 1. The setup consists of two ultrahigh vacuum chambers (pressure ≈ 2$\;\times 10^{-8}$ Pa), which are connected by a 15-cm-long glass tube with a diameter of 39 mm. The frequency of trapping lasers is approximately detuned 1.5 Γ from the D_2_ transitions (5^2^S_1/2_→5^2^P_3/2_) of ^87^Rb, where Γ = 2π × 6.07 MHz is the natural linewidth of the D_2_ line. The ^87^Rb atoms are trapped and cooled in the left chamber (source chamber) by utilizing the standard three-dimensional magneto-optical trap (3D-MOT) technique [14]. Then, the cold atoms are pushed to the right chamber (detecting chamber) and generate an atomic beam (see the gray areas in Figure 1a) because of the unbalanced forces generated by the laser in the longitudinal direction [15,16,17].

A second 3D-MOT technique is performed in the right chamber with a similar mechanism to the first one but with the three pairs of retro-reflected trapping lasers inclined by 45° with respect to the vertical direction. After the second MOT is loaded from the atom beam, a vertical 1064-nm Gaussian guiding laser, which is red-detuned with respect to the D-line dublett transitions of rubidium [18], is used to guide the cold atoms by the dipole potential. As shown in Figure 1a, the guiding laser can be expanded 2×~10× by a beam expander, and then reflected upwards by a reflecting mirror. It is propagated vertically through the chamber and then focused at the center of the atom cloud by a focusing lens, which has a focal length of 150 mm. Based on the intensity profile of the guiding laser in the experiment, the dipole potential distribution U_dipole_(r) can be calculated by U_dipole_(r) = (hδ/2) × ln[1 + (I(r)/I_s_)/(1 + 4δ^2^/Γ^2^)] [6], where δ is the laser detuning, I_s_ is the saturation intensity [19], and I(r) is the intensity distribution of the laser field. The calculated dipole potential distribution is presented in Figure 1b, which contributes to the comparison with the distribution of experimental guided atoms in the next section. Moreover, the fluorescence imaging technique is used to detect the profile of the atom cloud and count the number of cold atoms trapped by the guiding laser. A 780-nm detecting laser, with its frequency resonant to the D_2_ transitions of ^87^Rb, is coupled to one of the trapping lasers; thus, the fluorescence image of trapped atoms can be captured by a charge-coupled device (CCD) camera.

In contrast, a rigorous timing sequence of MOT1 and MOT2 is necessary to obtain the optimum experimental result. As shown in Figure 1c, during period t_1_, the cooling and repumping lasers of both MOTs are switched on, so atomic clouds are produced in both MOTs; during t_2_, MOT1 is switched off while MOT2 still works normally, implying that the source for MOT2 disappears, but the atomic cloud in MOT2 can still be maintained independently for some time. During t_3_, by switching MOT2 off, the releasing procedure of the atom cloud is recorded by the CCD camera, which operates at a frame-rate of 200 fps. Note that the guiding laser is always on in the atom guiding process to simplify the time sequence. This might affect the distribution of the atom cloud in MOT2, which will be discussed in the following section.

## 3. Results and Discussion

**Figure 2:**
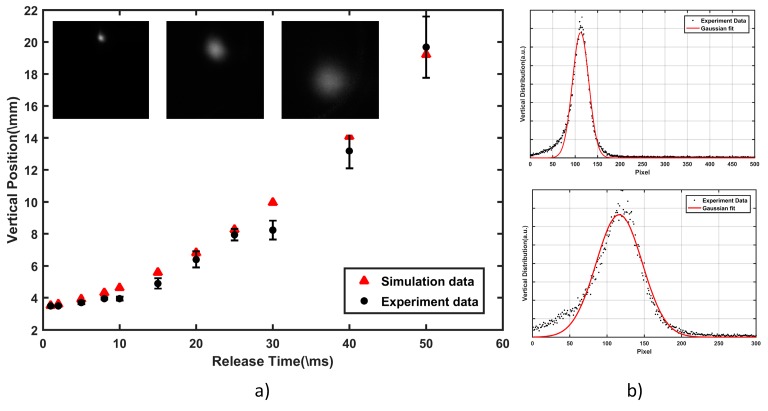
(**a**) Vertical position of free-fall atoms at varied release times. The insets are fluorescence images of the atom cloud in the detecting chamber, captured at 2, 10, and 40 ms after MOT2 is switched off. (**b**) Vertical distribution of released atom cloud at 2 and 5 ms after MOT2 is switched off. The red lines show the Gaussian fit of the experimental data.

The parameters of the atom cloud in MOT2 are important for the analysis of atom guiding and must be measured before the guiding experiment. During this step, the guiding laser is not necessary and is always switched off. And after captured by MOT2 in the detecting chamber, the atom cloud, which has a radius (σ) of about 133 μm and contains about 3.7 × 10^7^ atoms, is released and the free-fall images are recorded with varying release times, indicated in the insets of Figure 2a. Figure 2a also illustrates that the vertical position of the atom cloud is in agreement with the theory calculation deduced from the acceleration of gravity, which presents the guiding distance we achieved in the following experiment. Figure 2b shows that the vertical distribution of atoms in the situation approximately 2 and 5 ms after MOT2 is switched off. From the Gaussian shape expansion image, the temperature of the atom cloud is calculated to be about 16.65 μK based on the time-of-flight (TOF) method [20]. Theoretically, atoms with such a low temperature are sufficiently cold to be captured by the potential trap induced by the guiding laser [11].

**Figure 3:**
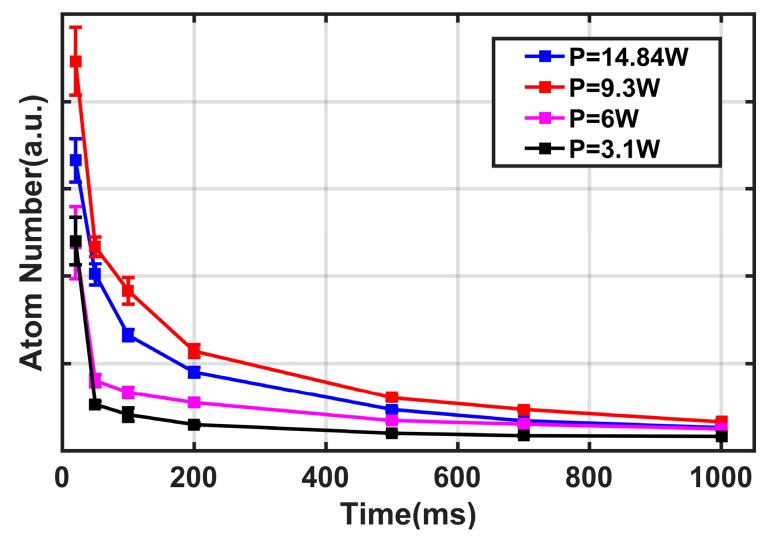
Dependence of number of guided atoms on time at different laser powers. Error bars on selected experimental points are representative of the standard error associated with our analysis method.

First, the beam waist diameter (2*w*_0_) of the guiding light was fixed at a constant value (40 μm), and the laser power used in this experiment was varied from 2 to 15 W, corresponding to the potential energy U_0_ at the center of the beam from 1.7 to 12.9 mK based on the theory in the previous section, to investigate the lifetime of the guided atoms within the potential trap. As illustrated in Figure 3, the number of atoms in the dipole trap attenuates with time as an approximately exponential function. This phenomenon can be explained by the fact that the repulsive force is derived from the reabsorption of emitted photons by the other atoms [21], cold collision [22], and light collisional losses [23]. Meanwhile, the increase in laser power can generate deeper potential traps, thus making the atoms remain longer in the trap. Generally, the time when the number of captured atoms reaches 1/e of the maximum is defined as the lifetime, and the lifetimes here are about 25, 50, 105, and 112 ms, respectively corresponding to laser powers of about 3.1, 6, 9.3, and 14.84 W. 

**Figure 4:**
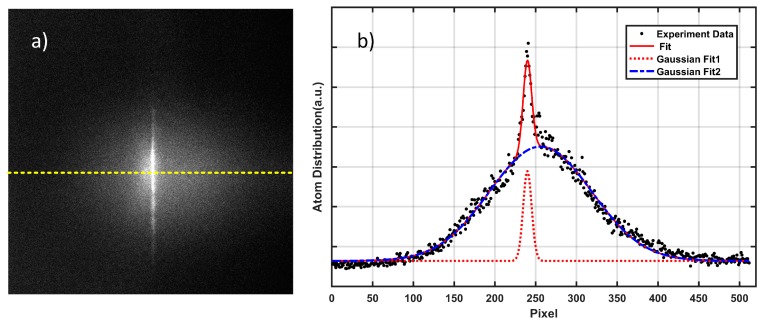
(**a**) Images of guided atoms released after 20 ms in the dipole trap. (**b**) Atom distribution indicated by the horizontal yellow dashed line in (**a**) over 512 pixels. The profile (red solid line) is fitted by two added Gaussian functions, where Fit1 (red dashed line) and Fit2 (blue dash dotted line) represent the guided and unguided atoms, respectively.

Secondly, we set the laser power to a constant value (8.9 W and 14.5 W) and investigated the influence of the laser beam waist (*w*_0_) on the guiding efficiency of the atoms. According to the experimental results mentioned above, the number of atoms in the dipole trap decreases with time. However, most atoms still remain in the trap 20 ms after MOT2 is switched off, so we compared the guiding efficiencies of the atoms in this case for different laser beam waists. The guiding efficiency here is defined as the relative value of the number (N_cap_) of guided atoms compared to the number (N) of all atoms trapped in MOT2 (N_cap_/N), and the guiding distance at 20 ms is around 3 mm. The fluorescence image of experimental guided atoms and the background-released atoms is shown in Figure 4. Numerical integration over five rows in Figure 4a is presented in Figure 4b, from which we can affirm that the distribution of guided and unguided atoms are well fitted by the Gaussian function.

**Figure 5:**
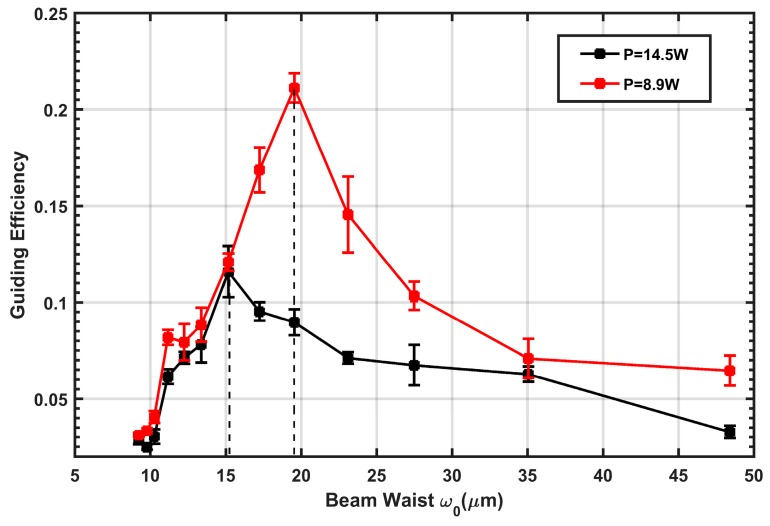
Dependence of guiding efficiency on beam waist at different laser powers, counted after MOT2 was released at 20 ms. Each experimental data point is an average over 10 measurements. The error bars correspond to standard deviation based on counting statistics.

As illustrated in Figure 5, the guiding efficiency increases dramatically with the beam waist and achieves the optimized value when the beam waist (*w*_0_) is around 15 μm and 20 μm, for the case of two guiding laser powers. However, as the beam waist increases further, the guiding efficiency slowly decreases. This experimental result can be approximately explained by the two counteracting trends: as the beam waist rises, the atomic ensemble has a larger interacting region with the guiding laser, so the number of guided atoms initially increases; however, the density of light intensity at the focusing point decreases at the condition of constant laser power, which causes the potential trap to decrease in depth. When the latter dominates, the guiding efficiency decreases.

The variation trend in the guiding efficiency with the beam waist is approximately in good agreement with the theoretical calculations in References [12,13], and the existence of an optimal beam waist is experimentally demonstrated here. Additionally, in the experiment, the optimal beam waist (*w*_0_) is maintained at around 15 μm and 20 μm, respectively, to which the corresponding U_0_/k_B_ is about 2.2 mK and 2 mK, for the two different laser powers employed in our experiment. This result is also valid according to the finding of References [12,13], that the optimal beam waist rises with the increase of U_0_/k_B_T. Here, T = 16.65 μK is the temperature of the atom cloud mentioned before. 

However, there still exists a notable disagreement: the ratio of the optimal beam waist to the atom cloud radius in the experiment is much smaller (*w*_0_/2σ ≈ 0.07, U_0_ ≫ k_B_T, the factor 2 results from the different definition of the waist of the guiding laser and the size of the atom cloud, see Equations (2) and (3) from Reference [11]) than the calculated prediction (*w*_0_/2σ ≈ 1 to obtain a guiding efficiency of about 75%, where U_0_/k_B_T = 4).

There are a few possible explanations for this. It should be noted that the calculation models from both references are based on the hypothesis that the initial position and velocity distribution are described by independent Gaussian functions. However, in our experiment, this hypothetical condition may be invalid because the guiding laser was constantly switched on from the beginning of the experiment. Compared to the initial atom cloud with a radius (σ) of 133 μm, the beam waist of the guiding laser in the experiment was so small (*w*_0_ varies from 10 to 50 μm, as shown by the x-axis of Figure 5) that the dipole trap generates a strong horizontal-compression force for the atoms. Thus, the momentum and position distributions of the initial cold atoms cannot be assumed as an ideal Gaussian function, but the effect of the guiding laser should also be considered, which implies that we should add the element of trapped atoms to the standard Gaussian distribution because of the dipole potential trap. Second, it should be noted that the imperfections of the experiment—such as the misalignment of 45°—can give rise to undesired reflections as well as affect the distribution of the atom cloud in the detecting chamber.

## 4. Conclusions

In this study, using a red-detuned Gaussian laser, we experimentally demonstrated the existence of an optimal laser beam waist for the guiding efficiency of cold atoms. Our experimental results demonstrate that, in the case that cold atoms are released from the MOT and then expanding ballistically in the red-detuned guiding potential trap, the guiding efficiency strongly depends on the beam waist of the laser. We successfully guided atoms for a distance of about 3 mm. The analysis of the experimental data shows that the guiding efficiency first increases with the beam waist radius and then peaks at around 20 μm, which is about 13 and 22% when the input laser powers are 8 and 14.85 W, respectively. However, as a consequence of the increasing guiding laser size, the laser intensity decreases and results in the weakening of the guiding effect. The comparison of our experimental results to the theoretical research by previous researchers is also qualitatively discussed, from which it can be confirmed that an optimal laser beam waist was realized in our research.

In contrast, we should also be aware of the disagreement between the calculation and our experimental results regarding the issue of the specific value of the optimal laser beam waist or the relationship between it and the atom cloud size. Therefore, further discussions are crucial. In follow-up investigations of the experimental imperfections, issues such as the reflection caused by the angle of incidence of the cooling laser should be addressed. Moreover, an accurate and comprehensive simulation model, in which the actual distribution of cold atoms when the guiding laser exists is taken into account, must be established. In addition, the influence of the laser beam waist on the guiding efficiency must be well interpreted. Finally, we would like to refer to the significance of our study, since in some applications the light field size is in the micron-scale, which is small compared to the dimensions of a cold atom cloud, such as that guiding cold atoms through hollow core fiber. Therefore, the study of the optimal mode field diameter is of great importance for optimizing the transferring efficiency.
